# Association between metabolically healthy overweight/obesity and gallstones in Chinese adults

**DOI:** 10.1186/s12986-023-00741-4

**Published:** 2023-03-31

**Authors:** Junlu Zhang, Liangli Chen, Keqing Shen, Jia Zhang, Yue Zhu, Qiaohua Qiao, Liying Chen

**Affiliations:** Department of General Practice, Sir Run Run Shaw Hospital, School of Medicine, Zhejiang University, Hangzhou, Zhejiang China

**Keywords:** Metabolic syndrome, Obesity, Gallstones

## Abstract

**Background:**

Metabolic syndrome and obesity are risk factors for gallstones. However, these two factors often occur together, and few studies have focused on the association between metabolically healthy overweight/obesity (MHOW/MHO) and gallstones. We hypothesized that MHO individuals would be associated with the prevalence of gallstones.

**Methods:**

This cross-sectional study included 125,668 participants aged 18–80 years at the Health Promotion Center of Run Run Run Shaw Hospital, Zhejiang University School of Medicine during 2017–2019 years. Each participant underwent a comprehensive health checkup. Gallstones were diagnosed by abdominal ultrasonography. Metabolically health was defined as not meeting the diagnostic criteria for metabolic syndrome (MetS). Obesity was measured by BMI. MetS and weight stratification were combined to classify the metabolism-obesity phenotypes. Logistic regression models were used to estimate adjusted odds ratios (ORs) and 95% CIs.

**Results:**

Among 125,668 participants, 5486 (4.4%) had gallstones. 21407 (17.0%) were MHOW individuals, and 3322 (2.6%) were MHO individuals. MHOW (OR 1.40; 95%CI: 1.29–1.53) and MHO (OR 1.80; 95%CI: 1.53–2.12) participants were at higher risk of gallstones and had larger and more numerous gallstones than metabolically healthy normal weight participants. Obesity, MetS, premenopausal women and advanced age were significantly associated with the prevalence of gallstones.

**Conclusions:**

MHOW/MHO individuals exhibited a higher risk of gallstones. In metabolically healthy individuals, the risk of gallstones increased with increasing BMI. Thus, obesity was associated with the prevalence of gallstones, even in relatively metabolically healthy adults.

**Supplementary Information:**

The online version contains supplementary material available at 10.1186/s12986-023-00741-4.

## Introduction

Gallstones are masses in the gallbladder or biliary tract caused by abnormally high levels of cholesterol or bilirubin in the bile. Gallstones are relatively common, accounting for about 10%–20% of the adult population worldwide, with a prevalence of 4.2%–23% in the Chinese population [1, 2]. Symptomatic gallstone disease can lead to serious and potentially life-threatening complications, such as acute cholecystitis, acute cholangitis and biliary pancreatitis. Gallstones are also associated with increased incidence and mortality of common chronic diseases, such as diabetes, hypertension, cardiovascular disease and gastrointestinal tumours [3–7]. Although the risk of gallstones is lower in China, the prevalence of gallstones is increasing steadily [2]. This is because a high-calorie, high-carbohydrate, low-fibre diet and lack of physical activity contribute to obesity and related metabolic disorders [1, 8].

Several studies have shown that age, gender, body mass index (BMI), non-alcoholic fatty liver, type 2 diabetes, and insulin resistance are associated with gallstones [9–11]. Obese people are at a higher risk of gallstones [12]. Metabolic abnormalities can also increase the risk of gallstones [13]. However, the two often occur together. This is because the effects of obesity on disease are mostly mediated through obesity-related metabolic abnormalities [14–16]. Therefore, it is more difficult to state whether there is an association between metabolically healthy obesity (MHO) and the prevalence of gallstones.

There are few studies on the association between MHO and gallstones. And studies by Su and Man have yielded different conclusions [17, 18]. They both classified the metabolism-obesity phenotypes in relation to metabolic markers and BMI, and compared the prevalence of gallstones between the groups. Su et al. concluded that MHO individuals were not significantly association with the prevalence of gallstones. Man et al. concluded that MHOW/MHO individuals were associated with an increased risk of gallstones.

Previous studies had small sample sizes (only 3190 in one of them), did not further stratify obesity, and never included the size and morphology of gallstones in the study. In this study, based on the previous literature, a larger amount of data was used to further stratify the obese population by combining the WHO obesity stratification and investigating the association between the prevalence of gallstones and the degree of obesity. In addition, this study further classified gallstones into single, multiple, mud-like, and filled as described by gallbladder ultrasound, and included the size of each type of gallstone for correlation analysis. The association between metabolically healthy obesity and gallstones was further argued.

Therefore, the main aim of this study was to investigate the association between MHO and the risk of gallstones in Chinese adults. This study combined metabolic syndrome (MetS) and BMI to classify the participants into metabolism-obesity phenotypes and to assess the differences in the prevalence of gallstones between the phenotypes. In addition, we examined whether the differences differed by age, gender, smoking and alcohol intake history.

## Subjects and methods

### Study population

**Fig. 1:**
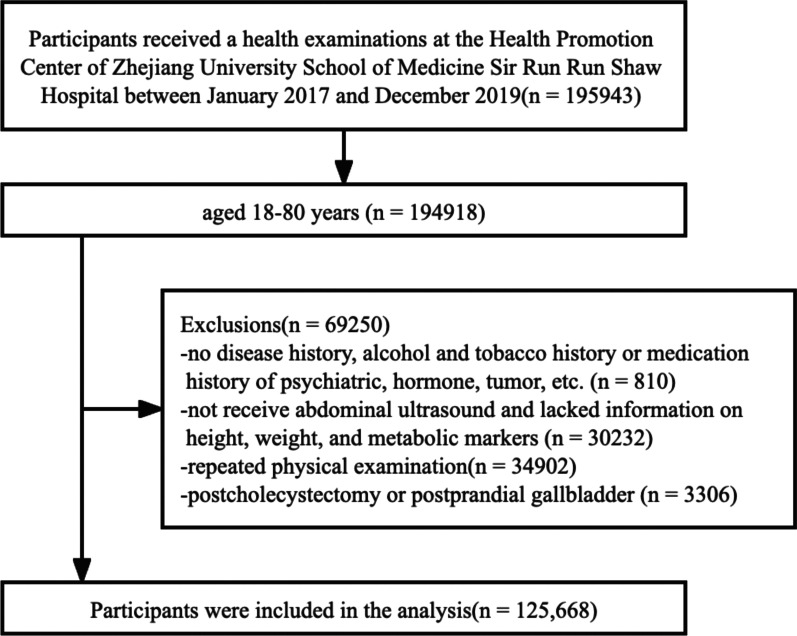
Flowchart of the study participants

The cross-sectional study included participants who voluntarily underwent checkups at the Health Promotion Center of Sir Run Run Shaw Hospital, Zhejiang University School of Medicine, from January 2017 to December 2019. Initially, we included adults aged 18–80 years (*n* = 194,918). 69,250 participants were excluded, of whom 810 participants had no disease history, alcohol intake and smoking history and had medication history of psychiatric, hormonal and oncological medication; 30,232 participants did not receive an abdominal ultrasonography and lacked information on height, weight and metabolic makers; 34,902 participants had a repeat checkups; and 3,306 participants had a cholecystectomy, or abdominal ultrasonography of a postprandial gallbladder. In the end, 125,668 participants were included in the analysis (Fig. 1).

### Data collection

Each participant underwent a comprehensive checkup. During the physical examination, medical, smoking and alcohol intake and medication histories were taken by trained general practitioners. Height, weight, waist circumference (WC) and systolic and diastolic blood pressure (SBP/DBP) were measured using standard protocol calibrated instruments and recorded by trained nurses. Fasting venous blood samples were collected from each participant, including fasting blood glucose (FBG), triglyceride (TG), total cholesterol (TC), high-density lipoprotein cholesterol (HDL-C), low-density lipoprotein cholesterol (LDL-C) and uric acid (UA). Fasting standard: more than 8 h and less than 16 h since the last food intake. Abdominal ultrasonography was performed by professional sonographers.

### Assessment of variables

Body weight was classified according to the BMI criteria proposed by the China Obesity Working Group in 2004 [19]. It was classified as underweight, normal weight, overweight and obese. Then combined with the WHO stratification criteria for obesity [20], the obese population was further classified into obese class I, obese class II and obese class III. MetS was based on the harmonized metabolic syndrome proposed by the International Diabetes Federation Task Force on Epidemiology and Prevention in 2009 [21]. The diagnostic criteria for weight stratification, obesity stratification and MetS were shown in Table 1. Participants were classified into eight phenotypes by combining BMI and MetS criteria*: Metabolically healthy underweight (MHU), metabolically healthy normal weight (MHN), metabolically healthy overweight (MHOW), Metabolically healthy obesity (MHO), metabolically unhealthy underweight (MUU), metabolically unhealthy normal weight (MUN), metabolically unhealthy overweight (MUOW) and metabolically unhealthy obesity (MUO). Then, the obese population was classified into metabolically healthy obese class I (MHO(I)), metabolically healthy obese class II (MHO(II)), metabolically healthy obese class III (MHO(III)), metabolically unhealthy obese class I (MUO(I)), metabolically unhealthy obese class II (MUO(II)), metabolically unhealthy obese class III (MUO(III)). (* In this manuscript, "metabolic health" only means "without metabolic syndrome" and "metabolically unhealthy" means "with metabolic syndrome").

**Table 1:** Diagnostic criteria for obesity and metabolic syndrome

Diagnose	Criterion	
BMI*		
Underweight		BMI < 18.50 kg/m^2^
Normal weight		BMI 18.50–23.99 kg/m^2^
Overweight		BMI 24.00–27.99 kg/m^2^
*Obese*		BMI ≥ 28.00 kg/m^2^
Obese class I		BMI 28.00–34.99 kg/m^2^
Obese class II		BMI 35.00–39.99 kg/m^2^
Obese class III		BMI ≥ 40.00 kg/m^2^
*MetS#*		
Elevated blood pressure		SBP ≥ 130 mmHg or DBP ≥ 85 mmHg or prior diagnosis of hypertension
Elevated blood glucose		FBG ≥ 100 mg/dL (5.6 mmol /L) or previous diagnosis of diabetes
Low HDL-C		HDL-C < 40 mg/dL (1.03 mmol/L) in men or < 50 mg/dL (1.29 mmol/L) in women or with previous diagnosis of hyperlipidemia
High TG		TG ≥ 150 mg/dL (1.70 mmol /L) or the use of fibrate antihyperlipidemic drugs
Abdominal obesity		WC ≥ 85 cm in men or ≥ 80 cm in women

*BMI = weight (kg)/height squared (m^2^)

^#^MetS = meet at least three of the above five criteria

The diagnosis of gallstones was based on abdominal ultrasonography. Participants were asked to fast for 8–16 h before the checkup. Gallstones were diagnosed by specialist sonographers. The ultrasonic diagnosis criteria of gallstones were: a stable hyperechoic mass appears in the cholecystic cavity, followed by a clear acoustic shadow, and the mass moves in the direction of gravity upon changing position [18]. Gallstones were classified as single gallstones, multiple gallstones (≥ 2), mud-like gallstones, and filled gallstones (gallbladder filled with mud-like stones) according to ultrasound description.

Other covariates: gender was categorized as male and female. Age was analyzed as a continuous variable. Smoking and drinking status were categorized into current and no smoking/drinking. Serum biochemical analyses including TC, LDL-C and UA were analyzed as continuous variables.

### Statistical analysis

All statistical analyses were performed using SPSS version 26.0 (SPSS Inc., Chicago, IL, United States). During the statistical analysis, the mean ± standard deviation was used for continuous variables, and ANOVA was used for significant differences between three groups and above; frequency (percentages/thousands of points) was used for categorical variables, and chi-square test was used for significant differences between groups. The multivariate logistic regression model was used to evaluate the odds ratios (OR) and 95% confidence intervals (95% CI) of gallstones in each metabolism-obesity phenotype, and build Model 1. Then, Age, gender, smoking and drinking history were adjusted, and build Model 2 and Model 3. P-trend was used to assess trends among ordinal categorical variables. In all statistical tests, a two-sided *P*-value < 0.05 was considered significant.

## Results

### Baseline characteristics

Of the 125,668 participants, 70,915 (56.4%) were male and 54,753 (43.6%) were female (Table 2). The mean age was 43.9 ± 11.9 years, 44.4 ± 11.9 years for males and 43.2 ± 12.0 years for females. Those with gallstones were 5486 (4.4% of the total), of which 3100 (56.5%) were males and 2386 (43.5%) were females (Table 2). The number of participants with MetS was 39,455(31.4% of the total) (Table 2), of which 29,495 (74.8%) were males and 9,960 (25.2%) were females. The mean BMI was 24.7 ± 3.16 kg/m2 for males and 22.4 ± 3.03 kg/m2 for females.

**Table 2:** Baseline characteristics of participants and those with or without gallstones

Characteristics	Overall (*n* = 125,668)	No gallstones (*n* = 120,182)	Gallstones (*n* = 5486)	*P* value
Age (years) †	43.9 ± 11.9	43.6 ± 11.9	50.5 ± 11.4	** < 0.001**
Gender (n, %)‡				
Male	70,915 (56.4%)	67,815 (56.4%)	3100 (56.5%)	0.906
Female	54,653 (43.6%)	52,367 (43.6%)	2386 (43.5%)	
Smoking (n, %)‡	2257 (1.8%)	2170 (1.8%)	87 (1.6%)	0.231
Drinking (n, %)‡	1687 (1.3%)	1626 (1.4%)	61 (1.1%)	0.129
MetS (n, %)‡	39,455 (31.4%)	36,917 (30.7%)	2538 (46.3%)	** < 0.001**
BMI (kg/m2) †	23.7 ± 3.3	23.7 ± 3.3	24.7 ± 3.3	**0.004**
WC (cm)†	82.5 ± 10.5	82.2 ± 10.5	85.9 ± 10.1	** < 0.001**
SBP (mmHg)†	121.3 ± 16.5	121.1 ± 16.4	126.4 ± 17.4	** < 0.001**
DBP (mmHg) †	72.7 ± 11.1	72.6 ± 11.1	75.1 ± 11.2	0.463
FBG (mmol/L) †	5.3 ± 1.1	5.3 ± 1.1	5.5 ± 1.4	** < 0.001**
TG (mmol/L)†	1.6 ± 1.4	1.6 ± 1.4	1.7 ± 1.3	0.809
HDL (mmol/L)†	1.3 ± 0.3	1.3 ± 0.3	1.2 ± 0.3	** < 0.001**
TC (mmol/L)†	4.8 ± 0.9	4.8 ± 0.9	4.9 ± 1.0	** < 0.001**
LDL (mmol/L)†	2.7 ± 0.8	2.7 ± 0.7	2.8 ± 0.8	** < 0.001**
UA (mmol/L)†	349.7 ± 90.6	349.5 ± 90.7	353.6 ± 88.1	** < 0.001**
*metabolic-obesity phenotypes (n, %)‡*				** < 0.001**
MHU	5410 (4.3%)	5315 (98.2%)	95 (1.8%)	
MHN	56,074 (44.6%)	54,352 (96.9%)	1722 (3.1%)	
MHOW	21,407 (17.0%)	20,453 (95.5%)	954 (4.5%)	
MHO	3322 (2.6%)	3145 (94.7%)	177 (5.3%)	
MUU	65 (0.1%)	62(95.4%)	3 (4.6%)	
MUN	8366 (6.7%)	7847 (93.8%)	519 (6.2%)	
MUOW	21,806 (17.4%)	20,429 (93.7%)	1377 (6.3%)	
MUO	9218 (7.3%)	8579 (93.1%)	639 (6.9%)	

Bold indicates that this difference was statistically significant in participants with or without gallstones

^†^Data are presented as the mean ± S.D

^‡^Data are presented as number (prevalence)

*BMI* body mass index; *WC* waist circumference; *SBP* systolic blood pressure; *DBP* diastolic blood pressure; *FBG* fasting blood glucose; *TG* triglyceride; *HDL* high-density lipoprotein; *TC* total cholesterol; *LDL* low-density lipoprotein; *UA* uric acid; *MHU* metabolically healthy underweight; *MHN* metabolically healthy normal weight; *MHOW*, metabolically healthy overweight; *MHO* metabolically healthy obesity; *MUU* metabolically unhealthy underweight; *MUN* metabolically unhealthy normal weight; *MUOW* metabolically unhealthy overweight; *MUO* metabolically unhealthy obesity

The numbers and characteristics of participants with or without gallstones were shown in Table 2. Participants with gallstones were older (gallstones: 50.5 years VS no gallstones: 43.6 years) and had a higher prevalence of MetS (gallstones: 46.3% VS no gallstones: 30.7%), higher BMI and WC (gallstones: 24.7 kg/m^2^ VS no gallstones: 23.7 kg/m^2^; gallstones: 85.9 cm VS no gallstones: 82.2 cm). Participants with gallstones had higher SBP, FBG, TC, LDL, UA and lower HDL, and the differences were statistically significant. Between the two groups, the differences in gender, proportion of smokers and drinkers, DBP and TG were not significant. The number of participants with each metabolic-obesity phenotype and the prevalence of gallstones were also listed in Table 2 (last 9 columns). The prevalence of gallstones increased with increasing BMI in both the with/without MetS. Detailed analysis was shown in Fig. 3.

**Fig. 2:**
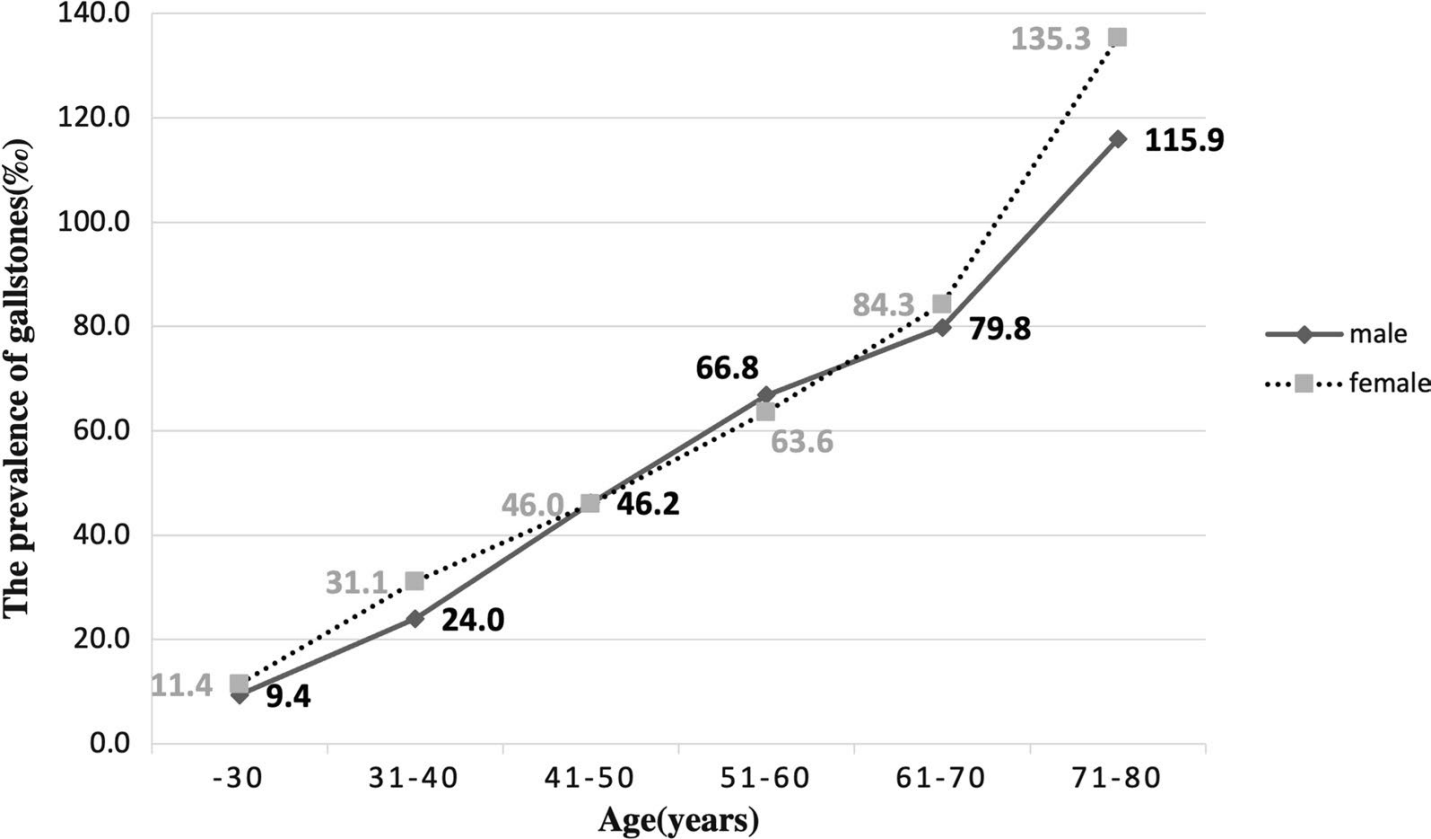
The prevalence of gallstones by gender and age. Participants: 125,668

The prevalence of gallstones by gender and age was shown in Fig. 2. The prevalence of gallstones increased with age in both males and females. In addition, at age 41–50 years, the prevalence of gallstones was 46.2‰ in males and 46.0‰ in females. There was no statistically significant difference between the two prevalence rates (*P* = 0.938). The prevalence of gallstones was higher in women than in men before the age of 45 (female: 27.7‰, male: 23.9‰, *P* = 0.01); after the age of 45, the gender difference in the prevalence of gallstones was not statistically significant (female: 65.1‰, male: 66.6‰, *P* = 0.245).

### Association between metabolism-obesity phenotypes and gallstones

**Fig. 3:**
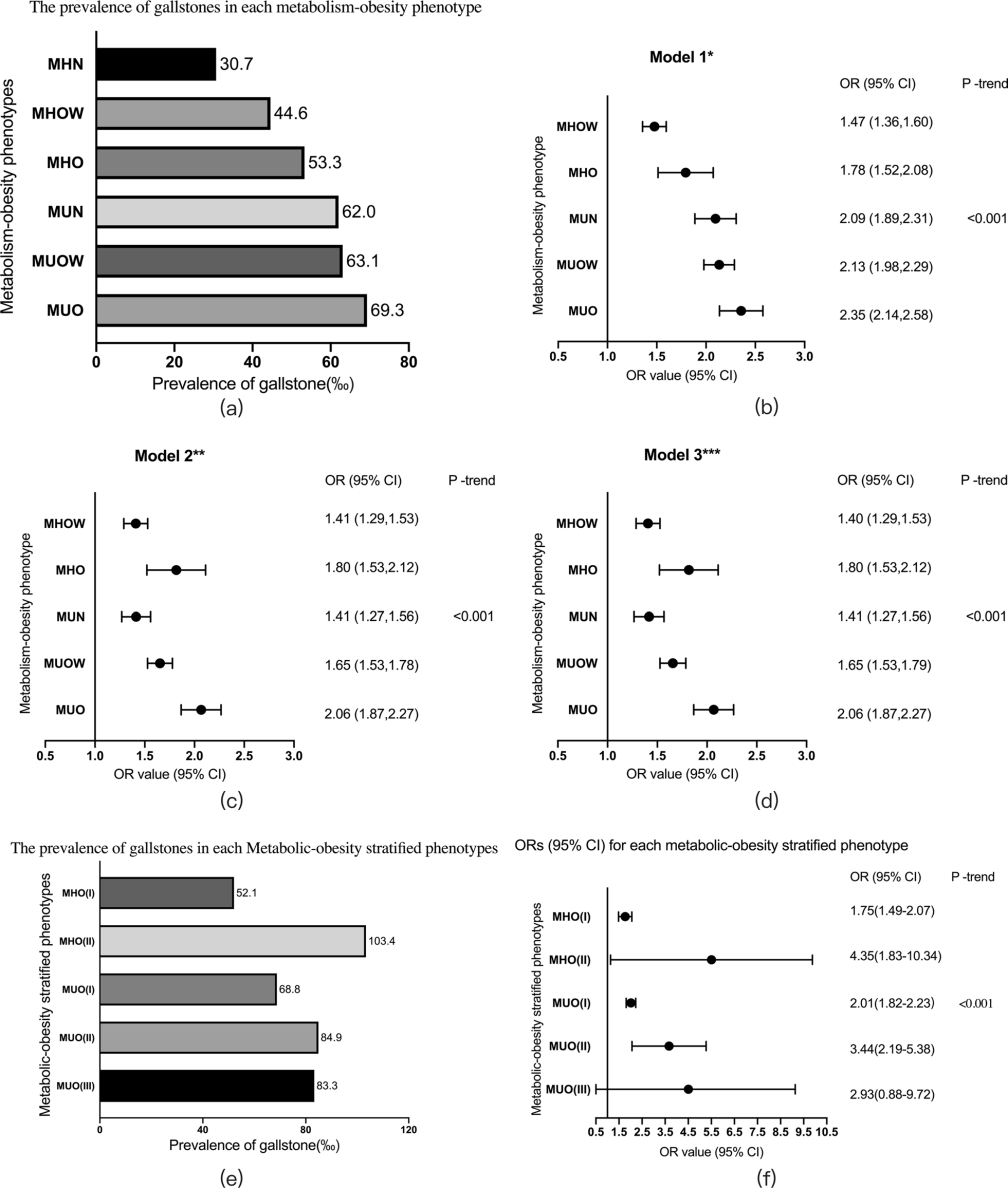
Association between metabolism-obesity phenotypes and gallstones. *Model 1 was not adjusted; **Model 2 was adjusted by age and gender; ***Model 3 was adjusted by age, gender, smoking status and drinking status. MHN, metabolically healthy normal weight; MHOW, metabolically healthy overweight; MHO, metabolically healthy obesity; MUN, metabolically unhealthy normal weight; MUOW, metabolically unhealthy overweight; MUO, metabolically unhealthy obesity. Participants: (**a–d**)120,193, (**e–f**)12,538

The prevalence of gallstones of each metabolism-obesity phenotype was shown in Fig. 3a (The prevalence of gallstones in the MUU group was not statistically different from that in any of the other groups after the chi-square test, with 65 participants and 3 gallstones. The MHU group was also excluded because it was an unmatched metabolically unhealthy group and was not the focus of this study). The prevalence of gallstones was higher in MHO (53.3‰) than in MHOW (44.6‰) and both were higher than MHN (30.7‰). The prevalence of gallstones was higher in metabolically unhealthy participants compared to metabolically healthy participants (MUN: 62.0%; MUOW: 63.1‰; MUO: 69.3‰).

Subgroup analyses were performed after logistic regression to determine the risk of gallstones in each phenotype. The results were listed in Fig. 3b–d. Model 1 was not adjusted, and it could be seen that when MHN was used as a reference, the ORs for each phenotype were MHOW: 1.47, MHO: 1.78, MUN: 2.09, MUOW: 2.13 and MUO: 2.35. In Model 2, gender and age were added for adjustment. In Model 3, gender, age, smoking and alcohol intake history were added for adjustment. The Ors were similar for each subgroup in both Model 2 and Model 3. The Ors of each phenotype were MHOW: 1.40, MHO: 1.80, MUN: 1.41, MUOW: 1.65 and MUO: 2.06 in Model 3. P-trend for each metabolism-obesity phenotype in the three models were all < 0.001.

The prevalence of gallstones for each metabolic-obesity stratified phenotype was shown in Fig. 3I (Since there were only two MHO(III), one of whom had gallstones, they were prone to large statistical errors. Therefore, it is not shown in Fig. 3e, f). The prevalence of gallstones was 52.1‰ for MHO(I) (*n* = 3262), 103.4‰ for MHO(II) (*n* = 58), 68.8‰ for MUO(I) (*n* = 8923), 84.9‰ for MUO(II) (*n* = 259), and 83.3‰ for MUO(III) (*n* = 36). Using model 3, logistic regression was performed for each group, and the ORs for each phenotype were shown in Fig. 3(f): MHO(I): 1.75, MHO(II): 4.35, MUO(I): 2.01, MUO(II): 3.44, and MUO(III): 2.93.

The association between each metabolic-obesity phenotype and the size and morphology of gallstones was shown in Table 3. For the prevalence of single/multiple gallstones, MHO was higher than MHOW, and MHN was the lowest. Although the prevalence of MHO was lower than that of the metabolically unhealthy participants, the difference between the groups was not statistically significant. For the size of single gallstones, MHN differed from MHO, MUOW, and MUO, MHOW differed from MHO, MUOW, and MUO, MHO differed from MUN and MUOW, and MUOW differed from MHO, and the differences were statistically significant. For the size of multiple/sediment-like gallstones, there were no statistically significant differences between the groups.

**Table 3:** Association between metabolic-obesity phenotypes and the size and morphology of gallstones

	Single (*n* = 2341)*	Single (cm)	Multiple (*n* = 2469)*	Multiple (cm)	Mud-like (*n* = 480)*	Mud-like (cm)	Filled (*n* = 98)*	Filled (cm)	Total (*n* = 5388)
MHN(n, ‰)	688 (12.3‰)a	1.34	804 (14.3‰)a	1.63	209 (3.7‰)a,b	2.33	21 (0.4‰)a	4.25	1722 (30.7‰)
MHO(n, ‰)	402 (18.8‰)b	1.41	469 (21.9‰)b	1.69	69 (3.2‰)a,b	2.31	14 (0.7‰)a,b	5.74	954 (44.6‰)
MHO(n, ‰)	84 (25.3‰)b,c	1.58	81 (24.4‰)b,c	1.73	5 (1.5‰)b	1.76	7 (2.1‰)b,c	3.75	177 (53.3‰)
MUN(n, ‰)	223 (26.7‰)c	1.38	244 (29.2‰)c	1.71	46 (5.5‰)a,c	2.11	6 (0.7‰)a,b,c	5.40	519 (62.0‰)
MUO(n, ‰)	659 (30.2‰)c	1.45	591 (27.1‰)c	1.56	92 (4.2‰)a,b,c	2.36	35 (1.6‰)c	4.40	1377 (63.1‰)
MUO(n, ‰)	285 (30.9‰)c	1.57	280 (30.4‰)c	1.60	59 (6.4‰)c	2.68	15 (1.6‰)b,c	4.09	639 (69.3‰)

*Different letters represent statistically significant differences between groups

*MHN* metabolically healthy normal weight; *MHOW* metabolically healthy overweight; *MHO* metabolically healthy obesity; *MUN* metabolically unhealthy normal weight; *MUOW* metabolically unhealthy overweight; *MUO* metabolically unhealthy obesity

Logistic regression was performed using Model 3 for each age group. The associations between all phenotypes and gallstones in different ages were shown in Additional file 1: Table S1. The other results of covariables were shown in Additional file 1: Table S2. As shown in Additional file 1: Table S1, the risk of gallstones increased in participants under 30 years of age only when their weight reached obesity (BMI ≥ 28 kg/m2) (MHO: OR 2.79, MUO: OR 3.90); in participants between 30 and 60 years of age, the risk of gallstones increased when their weight reached overweight (BMI ≥ 24 kg/m2); in participants older than 70 years, metabolically healthy obesity or MetS alone was not significantly associated with gallstones, and the risk of gallstones was associated with obesity combined with MetS (MUOW: OR 1.87; MUO: OR 1.99). As shown in Additional file 1: Table S2, the risk of gallstones was higher in women than in men up to the age of 50 years, with a gradual decrease with increasing age. And the gender difference became less significant after the age of 50 years. In this study population, there was no significant correlation between history of alcohol consumption or smoking and risk of gallstones.

## Discussion

In this cross-sectional study of checkup individuals, the prevalence of gallstones was higher in the participants with MetS compared to the participants without MetS. In both groups, the prevalence of gallstones increased with increasing BMI. This finding was more pronounced in the MH population. After adjusting for the effect of gender, age, and history of smoking and drinking, the OR of MHO was even higher than that of MUN and MUOW, but lower than that of MUO. After classifying gallstones by size and number, it was found that in the MH population, the size and number of gallstones, increased with increasing BMI. However, in the MU population, this result was not significant. This suggests that MHO is not a benign health status. This result applied to all participants aged 18–70 years. In people over 70 years, obesity combined with MetS increased the risk of gallstones. In addition, women before 50 years of age and advanced age are also associated with the risk of gallstones.

Many studies have shown that MetS-related factors increase the risk of gallstones [1, 22], and the association between obesity and gallstones has been confirmed in many epidemiological studies [8, 11]. However, obesity promotes insulin resistance, and insulin resistance causes a range of metabolic abnormalities that are determinants of MetS [23]. Thus, obesity and MetS often occur together, making it difficult to determine whether obesity per se is associated with gallstones. Currently, many studies have proposed multiple phenotypes combined with metabolic status and body weight to clarify the risk of each phenotype in different diseases [24, 25]. Assessing the risk of gallstones in all metabolism-obesity phenotypes could help to elucidate the role of obesity in the occurrence and development of gallstones.

The noteworthy finding of this study was among the MH participants, the prevalence of single/multiple gallstones increased with the increasing BMI (MHN: 30.7‰; MHOW: 44.6‰; MHO: 53.3‰), and there was a significant increase in size and number. Participants with the MHO phenotype had an even higher risk of gallstones than MUN and MUOW (MHO: OR 1.80; MUN: OR 1.41; MUOW: OR 1.65), but lower than MUO individuals (OR 2.06). The result held for participants before the age of 70, but was less significant after 70 years. It may be due to the fact that individuals over 70 years of age have more combined chronic diseases (e.g. type 2 diabetes, chronic kidney disease, chronic hepatitis, etc.) which interact with gallstones [22, 26–28].

There are three possible mechanisms by which obesity promotes the occurrence of gallstones. First, obesity can cause gallbladder motility disturbances. Among obese people, fasting and postprandial residual gallbladder volumes enlarge, and emptying delays [29]. Sustained supersaturation of cholesterol in bile enhances the absorption of cholesterol into gallbladder muscularis propria, reduces back diffusion of cholesterol to bile, and then causes gallstones [22]. Second, among obese individuals, cholesterol is hypersecretion from the liver into the bile due to upregulation of HMG-CoA reductase activity [30], leading to the formation of cholesterol gallstones. Cholesterol stones account for more than 90% of all gallstones [1]. Third, leptin and adiponectin, secreted by fat cells, have an additional link with gallstone formation. Obesity can increase leptin secretion, and it can regulate biliary lipid metabolism to promote the elimination of excess cholesterol stored in adipose tissue. Therefore, increased serum leptin can increase cholesterol secretion into bile, resulting in subsequent bile supersaturation with cholesterol and increased risk of gallstones [31]. Obesity can reduce serum adiponectin, which can lead to insulin resistance, then produce a series of metabolic abnormalities, and ultimately increase the risk of the occurrence of gallstones [32].

There are few studies on the correlation between MHO and gallstones. The survey by Su et al. concluded that metabolic unhealthy could lead to an increased risk of gallstones in people under 50 years old (MUO: OR 5.41, 95%CI 2.31–12.66), but obesity was not significantly associated with the risk of gallstones (MHO: OR 2.17, 95%CI 0.90–5.22) [17]. This difference can be partly explained by the small sample size of Su et al. (3,190 participants, 207 with gallstones). Man et al. found that overweight/obesity was associated with an increased risk of gallstones (MHOW: HR 1.37, 95%CI 1.07–1.75; MHO: HR 1.95, 95%CI 1.23–3.09) [18]. This is similar to the results obtained in our study.

In addition, we found that the prevalence of gallstones increased with age in both men and women. Although the overall prevalence of gallstones was higher in men, the prevalence of gallstones was higher in women before 50 years old, and was more significant after adjusting for MetS and BMI (≤ 30: OR 1.81; 31–40: OR 1.71; 41–50: OR 1.18). However, the gender difference became less significant after 50 years of age. This may be because estrogen enhances cholesterol synthesis (while decreasing bile acid synthesis) by upregulation of estrogen receptor α and the G protein-coupled receptor 30 [33]. After the age of 50, when women enter perimenopause, estrogen levels drop, and the risk of gallstones falls to a level similar to that of men. In our study, participants with gallstones had a higher BMI (24.7 kg/m^2^ for gallstones versus 23.7 kg/m^2^ for those without gallstones) and a higher prevalence of MetS (46.3% for gallstones versus 31.7% for those without gallstones), except in older and premenopausal women. This was consistent with the results of Amir et al. [34] and Francesco et al. [35].

The strength of this study was the large sample size, which thoroughly evaluated the correlation between each metabolism-obesity phenotype and gallstones in all age groups. But there were still some limitations. First of all, this was a cross-sectional study and a causal relationship between obesity/MetS and gallstones could not be established. Second, we did not distinguish between the type of gallstones (cholesterol or pigment) and the presence of gallbladder motility disorders. Third, the study failed to include confounding factors (e.g. dietary patterns, physical activity, HCV infection history, etc.) that might influence the outcomes. Fourth, smoking and drinking history was poorly collected in this study, with a larger proportion of the participants not completing their alcohol and tobacco use, resulting in a significant underestimation of alcohol and tobacco use. Fifth, the population in this study was from Zhejiang Province of China, with a predominance of yellow and Han Chinese, so the results may not be applicable to other races and ethnicities.

## Conclusions

In conclusion, in the cross-sectional study of participants in this chekups, individuals with MHOW/MHO had an increased risk of gallstones compared to MHN. In metabolically healthy individuals under 70 years, the risk of gallstones increased with increasing BMI. The results suggest that obesity per se can increase the risk of gallstones even in relatively metabolically healthy adults. In addition, metabolically unhealthy, advanced age and premenopausal women were also risk factors for gallstones in our study.

## Supplementary Information

**Additional file 1. Table S1.** Association between all phenotypes and gallstones in different ages group. **Table S2.** Association of other covariables with gallstones in different ages group.

## Data Availability

The raw data supporting the conclusions of this article will be made available by the authors, without undue reservation.
