# Supplementary material for: Association between metabolically healthy overweight/obesity and gallstones in Chinese adults
Source: Nutr Metab (Lond). 2023 Mar 31;20:20. doi: 10.1186/s12986-023-00741-4 (PMC10064513; doi:10.1186/s12986-023-00741-4)
Supplement: Supplementary file 1 — Additional file 1. Table S1. Association between all phenotypes and gallstones in different ages group. Table S2. Association of other covariables with gallstones in different ages group. [file 12986_2023_741_MOESM1_ESM.docx]

Supplementary Table 1 Association between all phenotypes and gallstones in different ages group

| **Age** | **MHN** | **MHOW** | **MHO** | **MUN** | **MUOW** | **MUO** |
| --- | --- | --- | --- | --- | --- | --- |
| **≤30**  OR (95%CI) | 1 | 1.20  (0.77,1.87) | 2.79  (1.50,5.17)* | 0.86  (0.21,3.53) | 1.75  (0.95,3.22) | 3.90  (2.36,6.44)* |
| **31-40**  OR (95%CI) | 1 | 1.84  (1.53,2.20)* | 2.85  (2.07,3.92)* | 1.87  (1.31,2.68)* | 1.79  (1.42,2.26)* | 2.80  (2.19,3.58)* |
| **41-50**  OR (95%CI) | 1 | 1.50  (1.30,1.73)* | 1.63  (1.20,2.21)* | 1.48  (1.20,1.82)* | 1.60  (1.38,1.85)* | 2.23  (1.86,2.66)* |
| **51-60**  OR (95%CI) | 1 | 1.20  (1.03,1.40)* | 1.50  (1.09,2.06)* | 1.30  (1.09,1.54)* | 1.59  (1.39,1.81)* | 1.69  (1.41,2.02)* |
| **61-70**  OR (95%CI) | 1 | 1.16  (0.90,1.50) | 1.95  (1.23,3.09)* | 1.37  (1.07,1.74)* | 1.49  (1.22,1.83)* | 1.81  (1.39,2.36)* |
| **>70**  OR (95%CI) | 1 | 1.27  (0.77,2.09) | 0.71  (0.21,2.40) | 1.45  (0.92,2.27) | 1.87  (1.29,2.73)* | 1.99  (1.24,3.18)* |

*P < 0.05

MHN, metabolically healthy normal weight; MHOW, metabolically healthy overweight; MHO, metabolically healthy obesity; MUN, metabolically unhealthy normal weight; MUOW, metabolically unhealthy overweight; MUO, metabolically unhealthy obesity.

Participants: 120193

Supplementary Table 2 Association of other covariables with gallstones in different ages group

| **Age** | **Gender** | **Age** | **Smoking** | **Drinking** |
| --- | --- | --- | --- | --- |
| **≤30**  OR (95%CI) | 1.81  (1.31,2.51)* | 1.09  (1.02,1.16)* | 0.00  (0.00, ~) | 2.52  (0.34,18.60) |
| **31-40**  OR (95%CI) | 1.71  (1.48,1.99)* | 1.09  (1.06,1.11)* | 1.68  (0.95,2.99) | 0.64  (0.29,1.38) |
| **41-50**  OR (95%CI) | 1.18  (1.06,1.32)* | 1.05  (1.03,1.07)* | 0.80  (0.51,1.24) | 1.04  (0.64,1.67) |
| **51-60**  OR (95%CI) | 1.03  (0.92,1.14) | 1.04  (1.02,1.05)* | 0.74  (0.48,1.13) | 0.70  (0.41,1.20) |
| **61-70**  OR (95%CI) | 1.07  (0.92,1.24) | 1.05  (1.02,1.08)* | 0.84  (0.38,1.85) | 0.45  (0.15,1.34) |
| **>70**  OR (95%CI) | 1.20  (0.91,1.58) | 1.08  (1.03,1.13)* | 0.68  (0.14,3.32) | 2.52  (0.61,10.35) |

*P < 0.05

Participants: 120193
